# Review on Coupled Thermo-Hydraulic Performance of Nanofluids and Microchannels

**DOI:** 10.3390/nano12223979

**Published:** 2022-11-11

**Authors:** Yuwei Wang, Jie Yu, Cong Qi, Wenjie Zhang

**Affiliations:** School of Low-Carbon Energy and Power Engineering, China University of Mining and Technology, Xuzhou 221116, China

**Keywords:** nanofluids, microchannel, thermal conductivity, viscosity, heat transfer enhancement

## Abstract

As electronic components continue to be miniaturized, the heat flux density continues to increase. Scholars have proposed the use of microchannel heat sinks (MCHS) to dissipate heat from devices with high heat flux density, and have pointed out that the heat dissipation capability of MCHS can be improved in two ways: using nanofluids with high thermal conductivity and optimizing the structure of MCHS. In this paper, the thermophysical parameters and thermo-hydraulic performance of nanofluids in microchannels are reviewed. Improving the heat dissipation of MCHS is analyzed and discussed in terms of both thermal properties and flow properties, respectively.

## 1. Introduction

With the miniaturization of electronic devices, the integrated density of transistors is increasing. At present, transistors have been developed to the nanometer level. Nano-scale quantum dot thermoelectric transport [1] and molecular-scale thermoelectric devices [2] have been proposed. Single-molecule junction is the most basic research unit. At present, some scholars have studied the thermal power of the single-molecule junctions of some materials (such as metal [3], oligomeric (styrene-acetylene) derivatives [4], carbon nanotubes and graphene [5,6]). The thermoelectric measurement platform using liquid eutectic gallium-indium (EGaln) to obtain large amounts of data was proposed by Park et al. [7]. Heat dissipation and thermoelectric effects in molecular junctions have also received extensive attention [8]. Therefore, it is urgent to find an efficient cooling method for micro devices to reduce energy consumption, improve equipment efficiency and increase service life [9,10].

Most conventional fluids have low thermal conductivity and poor thermal performance, such as water, ethylene glycol (EG) and oil. In 1995, Choi et al. [11] proposed to disperse metal nanoparticles into conventional fluids to make nanofluids and found that the thermal conductivity increased. Therefore, applying nanofluids to micro-nano scale heat dissipation can effectively improve the heat-dissipation effect [12].

Tuckerman and Pease [13] were the first to put forward the concept of microchannel heat sinks (MCHS) in 1981 and designed a water-cooled MCHS for heat transfer at a heat flux density of 790 W/cm^2^. It was found that the maximum temperature at the bottom was 71 °C higher than the water temperature at the inlet, and MCHS effectively improves the cooling effect. MCHS have the characteristics of small size, compact structure and good heat transfer, which can effectively solve the problem of the high heat flux density of tiny electronic components.

However, with the addition of metal nanoparticles, the viscosity of the nanofluids increases, resulting in a higher pressure drop and weaker flow properties in the microchannel heat sink, affecting the overall performance of the MCHS [14]. In addition, the structure and arrangement of the channels also affect the heat-dissipation effect of the MCHS [15]. 

Most researchers have reviewed the thermal properties of nanofluids and the effect of microchannel structure on the heat-dissipation effect of MCHS separately. Few researchers have investigated the effect of the combination of both on the flow and heat transfer characteristics of MCHS.

This paper is divided into three main parts. The first part mainly introduces the preparation method, physical properties and methods of passively enhancing heat transfer. The second part presents the state of research on the influence of different microchannel shapes, structures and distributions on the performance of MCHS in recent years. The third part describes the flow and heat transfer characteristics of different nanofluids and fluid flow states in different structures of MCHS. This paper provides a review of improvements in the heat-dissipation capacity of MCHS in terms of fluid properties and MCHS structure, respectively, providing a clearer direction for subsequent research.

## 2. Nanofluids

This section reviews and summarizes the methods of preparing nanofluids, the measurement methods of stability, the factors influencing physical properties (thermal conductivity, viscosity, surface tension and contact angle), as well as explaining the mechanisms by which nanofluids enhance heat transfer and reviewing methods for enhancing passive heat transfer in nanofluids.

### 2.1. Preparation

**Figure 1:**
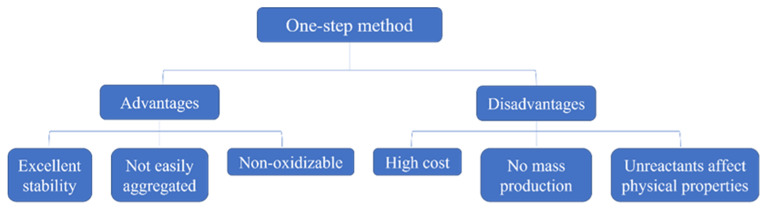
Advantages and disadvantages of the one-step method.

**Figure 2:**
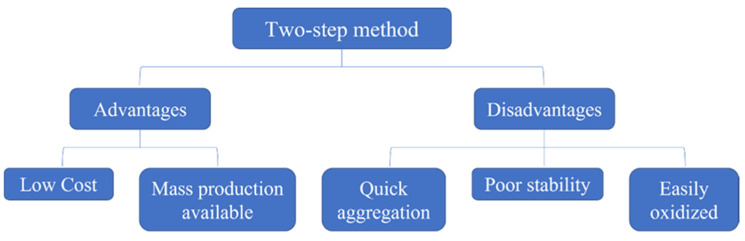
Advantages and disadvantages of the two-step method.

The performance of nanofluids is closely related to the preparation method. Common preparation methods are one-step and two-step methods [16,17,18]. The one-step and two-step methods each have advantages and disadvantages, as shown in Figure 1 and Figure 2. The appropriate method should be chosen for the preparation of nanofluids [19].

### 2.2. Stability

The stability of nanofluids is important for their thermal properties. Factors such as nanofluids preparation method [20], nanoparticle concentration [21], nanoparticle type, surfactant type, surfactant concentration, pH [22], sonication type and time and sonication power can affect the stability of nanofluids [23]. Many approaches are available to assess the stability of nanofluids, including sedimentation observation [24], transmittance method based on ultraviolet spectrophotometer [25], Zeta potential measurements [26], and the 3ω method [27]. 

#### 2.2.1. Settlement Observation Method

Ali et al. [20] used the settlement observation method to find the dispersed deposition of nanofluids prepared by the two-step method controlling the ultrasonic water-bath temperature below 30 °C and flocculent deposition by the conventional two-step method.

#### 2.2.2. Transmittance Method Based on UV Spectrophotometer

Cacua et al. [25] discovered that nanofluids with a critical micelle concentration of sodium dodecylbenzene sulfonate as the surfactant are more stable, while those without surfactant and those with cetyltrimethylammonium bromide as the surfactant are less stable, using the transmittance method based on a UV spectrophotometer.

#### 2.2.3. Zeta Potential Measurement

Zeta potential measurement is a method for assessing stability based on the DLVO theory [22]. A Zeta-potential greater than ±30 mV is considered stable, and within ±15 mV, it is considered unstable [24]. Parsa et al. [26] used the Zeta-potential method to conclude that nanofluids prepared using the one-step method are significantly higher than those prepared using the two-step method.

Many current studies combine qualitative assessment methods with quantitative methods [28], such as the transmittance method and Zeta-potential measurement method [29], to describe the stability of nanofluids in a more objective and intuitive way [30,31].

### 2.3. Thermal Conductivity

The thermal conductivity of nanofluids is a significant physical characteristic; the higher the thermal conductivity of the nanofluids, the better the heat-transfer performance. It has been found that the thermal conductivity of nanofluids is related to the type and size of nanoparticles, base fluid, concentration, temperature and pH. Table 1 summarizes the thermal conductivity of common nanofluids. It can be observed that the thermal conductivity improves with the increasing concentration of the nanofluids.

Soltani et al. [43] discovered that the thermal conductivity of engine oils decreases with the increasing temperature. However, the higher the temperature, the higher the thermal conductivity of the nanofluids, with the largest increase, by 5.63%, for *φ* = 0.2% MWCNTs-oil nanofluids from *T* = 20 °C to *T* = 60 °C.

Zhang et al. [49] experimentally investigated the effect of pH on the thermal conductivity of TiO_2_-H_2_O nanofluids. It was concluded that pH = 6.5 is the isoelectric point and the further the pH is from 6.5, the higher the thermal conductivity; pH = 2 and 10 have the highest thermal conductivity of the nanofluids.

Jeong et al. [50] examined the effect of the shape of nanoparticles on the thermal conductivity of ZnO nanofluids. The thermal properties of the nearly rectangular nanoparticles were more significantly enhanced, with an 18% rise in thermal conductivity.

### 2.4. Viscosity

The viscosity of nanofluids increases with the addition of nanoparticles. Pumping power and pressure drop are related to the viscosity of the fluid, which is an important physical parameter affecting the flow characteristics [51], and, therefore, the study of nanofluid viscosity is necessary. Existing studies revealed that nanofluids’ viscosity can be affected by temperature, nanoparticle size and shape and concentration, nanofluids preparation methods [52] and pH.

Li et al. [53] prepared and evaluated the viscosity for each mass fraction of ZnO-EG nanofluids at different temperatures. Karimipour et al. [54] analyzed the relationship between temperature and nanofluid viscosity through experiments and numerical simulations. They both conclude that viscosity decreases with increasing temperature.

**Figure 3:**
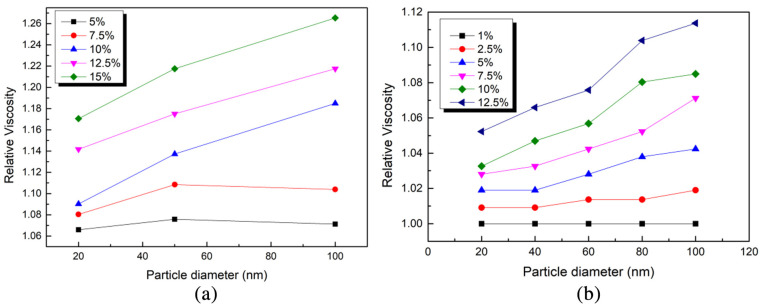
Effect of nanoparticle size on viscosity [56]: (**a**) Al_2_O_3_; (**b**) ZnO.

Agarwal et al. [55] found that the dynamic viscosity of paraffinic alumina nanofluids with different particle sizes (13 nm, 15 nm) decreases with increasing nanoparticles. Figure 3 is the conclusion drawn by Hu et al. [56] using SEM images and DLVO theory. With the change in particle concentration, the change trend of viscosity with particle size can be divided into three trends: decreasing, unchanged and increasing. As the size of the nanoparticles changes, the contact area between the nanoparticles and the base fluid changes, resulting in changes in interfacial resistance and flow layers, which, in turn, affect the viscosity of the nanofluid.

The relationship between volume fraction and viscosity of ZnO-Ag (1:1)-H_2_O hybrid nanofluids was experimentally investigated by Ruhani et al. [57]. Li et al. [58] and Hu et al. [38] researched the relationship between the viscosity and concentration of CuO-H_2_O and TiO_2_-H_2_O nanofluids, respectively. Both concluded that as the concentration of the nanofluids increases, the viscosity increases.

### 2.5. Surface Tension and Contact Angle

#### 2.5.1. Surface Tension

The addition of nanoparticles also alters the surface tension of the base fluid, affecting surface-tension-driven convective flow and heat transfer [58]. Kim et al. [59] revealed that incorporating Al_2_O_3_ nanoparticles into alcohol-based nanofluids resulted in an average increase in surface tension by 3.4%. Ilyas et al. [60] investigated the effect of concentration and temperature on the surface tension of a graphene nanoplate-based saline nanofluids using the pendant-drop method. Based on previous studies, it can be concluded that surface tension decreases with the addition of nanoparticles, decreases with increasing temperature and that different base fluids also change the surface tension. However, there are some inconsistent results for the effects of concentration and surfactant and further research is needed [61].

#### 2.5.2. Contact Angle

The contact angle is a parameter used to indicate the ability of a liquid to make contact with a surrounding solid surface [62]. The concentration, size and shape of nanoparticles in a nanofluid affect the size of the contact angle and, thus, change the wettability [63]. Huminic et al. [64] experimentally investigated the effect of solid (aluminum, copper and stainless steel) surfaces on the contact angle of nanofluids, using the Wilhelmy plate technique. It can be concluded that the wettability of nanofluids is superior to that of base fluids and that the contact angle of the nanofluids decreases with increasing temperature and concentration.

### 2.6. Methods of Passively Enhancing Heat Transfer

At present, improving the passive heat transfer of nanofluids has also been widely studied [65,66]. Changing the size [67,68], shape [69] and concentration [70] of the nanofluid can increase the thermal properties of the nanofluid and, thus, improve its thermal performance in the cavity.

In addition, one of the most common methods to enhance the heat-transfer capacity of nanofluids in a cavity is to alter the cavity structure to increase the interference of the nanofluids, disrupt the flow boundary layer and enhance heat transfer [71]. Table 2 lists some of the shape and structure optimizations.

Wang et al. [77] found that adding a magnetic field would lead to the formation of more vortices on the working surface and stronger heat transfer. Table 3 summarizes the influence of external field on the thermal properties of nanofluids.

Based on previous studies, it can be concluded that reasonable changes to the cavity structure and the addition of a suitable external force field can lead to an improvement in the thermal properties of nanofluids.

## 3. Microchannel Heat Sinks (MCHS)

The heat-transfer capacity of MCHS is mainly influenced by the structure and distribution of the microchannels, the thermal capacity of the solid medium and the thermal capacity and flow state of the fluid medium. Microchannels can be optimized by changing the channel shape, and adding ribs and pin-fins to the microchannel and secondary flow channel, etc. [87]. This section summarizes the influence of physical structure on MCHS performance in recent years from four main perspectives: the shape of and variation in the cross-section of the microchannels, the shape of the ribs and cavities, the shape of the pin fins and the form of the distribution of the channels.

### 3.1. Thermal-Hydraulic Properties of Different Microchannel Shapes in MCHS

**Figure 4:**
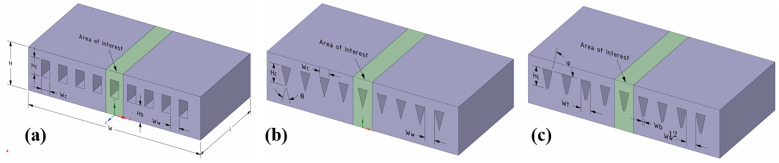
3D models of different channel cross-section shapes [88]: (**a**) rectangular channel; (**b**) triangular channel; (**c**) trapezoidal channel.

Figure 4 lists common channel shapes. Kose et al. [88] concluded that MCHS with rectangular channels (RMCHS) have the best thermal-hydraulic performance; the pump power can be reduced by 17% and 40% compared to trapezoidal and triangular shapes, respectively. Lv et al. [89] and Parlak et al. [90] found that microchannels with high aspect ratios have better heat dissipation and lower pressure drops, and that channel thickness and spacing affect the thermal resistance.

**Figure 5:**
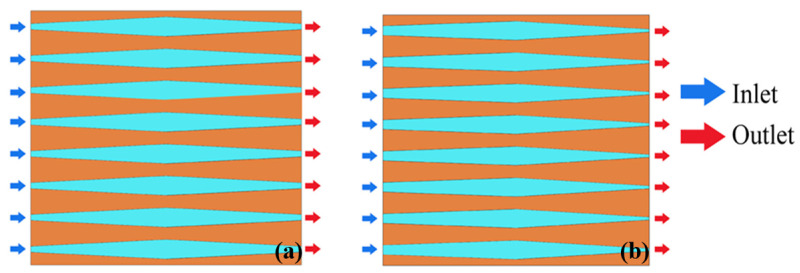
Microchannels with section width [91]: (**a**) symmetrical DCMCHCS; (**b**) asymmetric DCMCHCS.

Some researchers have further investigated the effect of rectangular variable section microchannels on the performance of MCHS. The thermal performance of microchannel-width divergence–convergence microchannel heat sinks (DCMCHS) [91] has been studied, as shown in Figure 5. It was obtained that the smaller the inlet-to-center-width ratio of symmetrical DCMCHS, the lower the thermal resistance and the higher the pressure drop. However, the study of microchannel-width-convergence MCHS [92] found that their thermal performance and that of asymmetrical DCMCHS both increase as the ratio of outlet-to-inlet width decreases.

**Figure 6:**
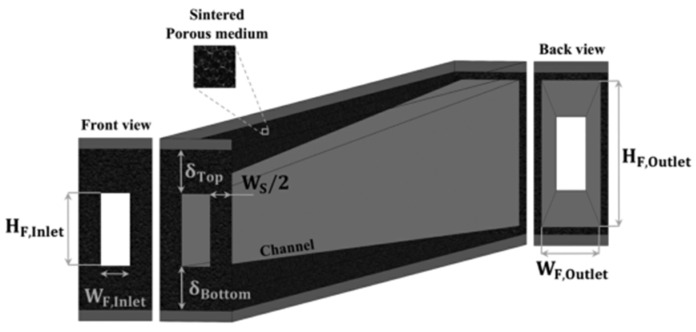
Highly divergent microchannel model [93].

Hajmohammadi et al. [93] found that channel height divergence can improve the thermal performance of MCHS, as shown in Figure 6. However, the improved performance of the MCHS is achieved by consuming more energy.

**Figure 7:**
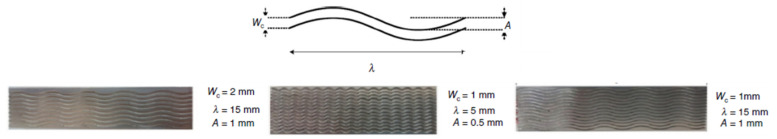
Wavy microchannel model [94].

Sajid et al. [94] carried out the waveform optimization of rectangular channels, as shown in Figure 7, and found that the effect of wavelength on the *Nu* is more significant than the effect of microchannel width. Khan et al. [95] further investigated the effect of base wavy shape on MCHS performance and found that a maximum thermal performance factor of 2.2 can be achieved.

### 3.2. Thermal-Hydraulic Properties of MCHS with Different Rib and Cavity Shapes

**Figure 8:**
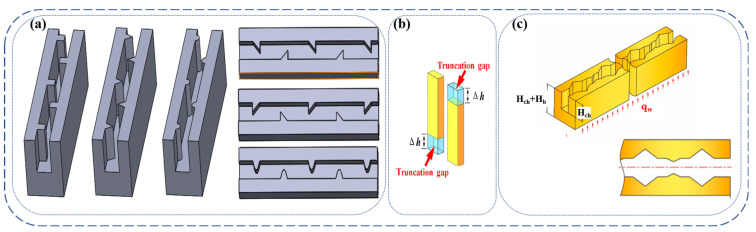
Models of different shapes of ribs and cavities: (**a**) shape of ribs [96]; (**b**) shape of truncated ribs [97]; (**c**) structure of ribs and cavities [98].

Based on the above, it is clear that variable cross sections can affect the performance of MCHS and, therefore, ribbed and cavitated microchannel structures have been proposed. The ribs will cause the flow boundary layer and the thermal boundary layer to be interrupted, which enhances heat transfer. Figure 8a [96] summarizes the common rib shapes and Figure 8b [97] is the truncated rib structure. The fluid in the cavity will form vortices to reduce the pressure drop. Therefore, a structure combining the ribs and cavities is proposed, as shown Figure 8c [98]. Table 4 summarizes the recent studies on the effects of ribs and cavities on MCHS.

### 3.3. Thermal-Hydraulic Properties of MCHS with Different Pin-Fin Shapes

**Figure 9:**
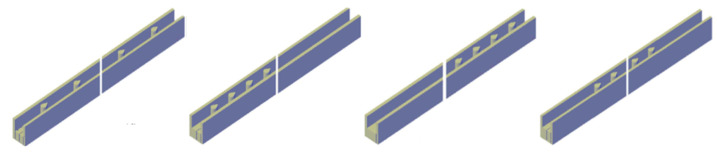
Distribution types of pin fin in channel [107].

The addition of pin fins in the microchannel can continuously cause the destruction and re-formation of the flow boundary layer, and also enhance the fluid perturbation to fully mix. Pin-fin shapes, sizes and distribution can cause different disturbance and mixing effects. common pin-fin shapes include square, circular, triangular and teardrop [106]. Figure 9 illustrates the different distribution types.

Table 5 lists the effects of pin fins on MCHS. Pin fin, rib and cavity can enhance heat dissipation. Combining them to study the comprehensive effect is also a hot spot of current research.

**Figure 10:**
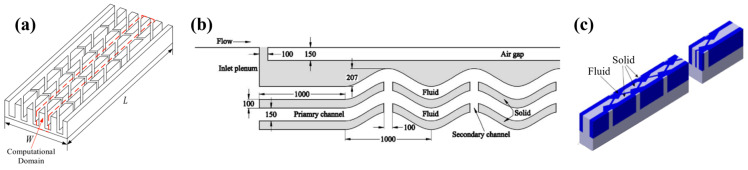
MCHS with secondary channels: (**a**) trapezoidal fins [115]; (**b**) wavy microchannel [117]; (**c**) microchannel with cavities and ribs [118].

Based on the above studies, the MCHS with secondary channels was proposed and investigated. Figure 10a,b show the MCHS with secondary channels formed by trapezoidal fins and parallelogram fins, respectively. It was found that the width ratio of secondary to primary flow channels has the greatest impact on the performance of MCHS [115,116]. The comparison of the influence of secondary flow caused by differently shaped pin fins needs further study.

Ma et al. [117] brought a secondary flow structure into the wavy microchannels, as shown in Figure 10b. This structure increases the surface area and creates an evolving thin evaporating liquid film, which are the main reasons for the enhanced heat dissipation, but also a larger pressure drop.

Japar et al. [118] introduced secondary channels into the MCHS with ribs and cavities, as shown in Figure 10c, and found that the thermal boundary layer undergoes a process of interruption, mixing and then re-formation, resulting in the enhanced performance of the MCHS.

### 3.4. Thermal-Hydraulic Performance of MCHS with Different Distributions

The different distributions of the microchannels will mainly affect the uniformity of the temperature and the flow characteristic. Table 6 shows the influence of microchannel distribution on the thermal-hydraulic performance of the MCHS. It can be observed that the thermal performance of the MCHS is enhanced with increasing contact area between the fluid and the heat surface as well as with increasing fluid disturbance [119].

In addition to single-layer microchannel distributions, there are also double-layer distributions. Zhou et al. [125,126] discovered that the maximum temperature and temperature rise of the double-layer MCHS (DL-MCHS) base plate are reduced, and the thermal resistance is decreased. Derakhshanpour et al. [100] found that ribbed DL-MCHS have a 30–60% higher convective heat-transfer coefficient than ribbed SL-MCHS. 

**Figure 11:**
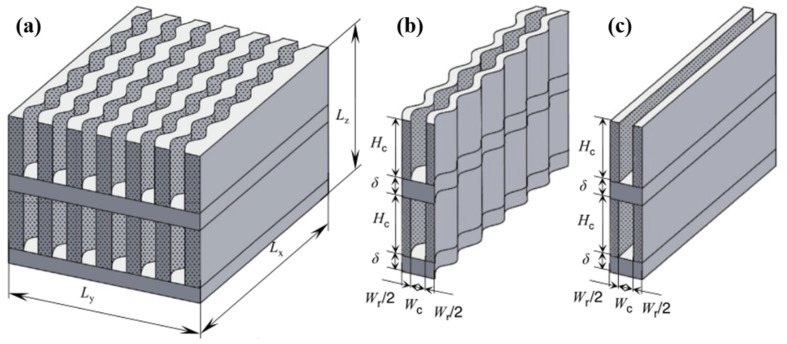
DL-MCHS structural model [128]: (**a**) upper layer wavy and lower layer straight; (**b**) double layer wavy; (**c**) double layer straight.

Further parametric and structural optimization of the DL-MCHS has been carried out by several researchers. DL-MCHS with a smaller channel width in the upper layer and a larger channel width in the lower layer can reduce the thermal resistance and enhance *Nu* [127]. As shown in Figure 11, the DL-MCHS with both upper and lower layers having wavy channels have low thermal resistance and induce more Dean vortices, allowing the sufficient mixing of hot and cold fluids for optimal cooling capacity [128].

## 4. Thermo-Hydraulic Performance of Nanofluids in MCHS

The heat dissipation capacity of the MCHS is closely related to the thermal properties and flow form of the working fluid. Nanofluids are used as working fluids in MCHS frequently due to their good thermal properties. This section provides a review of the effect of different nanofluids and flow states on the heat dissipation of MCHS. Table 7 summarizes the flow and heat-transfer performance of different MCHS structures using nanofluids in recent years.

The form of the flow of the fluid is also one of the most important elements affecting the heat-transfer performance of MCHS. Zhang et al. [138] examined the heat-dissipation capability of parallel-flow and counter-flow MCHS through numerical simulations, and found that the thermal performance of counter-flow MCHS is superior to that of parallel-flow MCHS. The thermal performance of parallel-flow and counter-flow DL-MCHS was also studied and it was found that counter flow has better thermal performance [126]. 

Awad et al. [139] found that the average temperature of jet-impacted MCHS is lower. Xu et al. [140] researched the thermal performance of MCHS driven by the square wave, triangular wave, sawtooth wave and sinusoidal pulsating flows of nanofluids, and the Nu can be increased by 16.5%. Mohammadpour et al. [141] used an algorithm to investigate the effect of dual synthetic jet parameters on the performance of MCHS nanofluids and found that the thermal conductivity could be increased by 53.99% for isotropic driving.

## 5. Conclusions and Prospects

This paper reviewed the physical properties of nanofluids, the effect of microchannel structures on the thermal performance of MCHS and the effect of different nanofluids and flow states on the thermal dissipation of MCHS, allowing the following conclusions to be drawn:

(1) The thermal conductivity increases with increasing concentration and temperature. The viscosity decreases with increasing temperature and increases with increasing concentration, and the viscosity varies with nanoparticles in relation to the concentration. The surface tension decreases with increasing temperature and the contact angle decreases with increasing temperature and concentration.
(2) Optimization of the cavity structure and the incorporation of external fields can improve passive heat transfer in nanofluids.
(3) Rectangular channels have the best MCHS performance, and variable channel cross-sections can improve heat dissipation. Adding ribs will destroy the boundary layer and increase the pressure drop; cavities will form vortices and reduce the pressure drop; pin fins will cause secondary flow, all three, for the microchannel structure, will increase the the heat dissipation of the MCHS.
(4) The combination of a high thermal-performance nanofluids and an optimized MCHS structure allows for the efficient cooling of the MCHS. A suitable fluid flow state can also improve the cooling performance of MCHS more significantly.

Most of the available findings are that the high thermal-conductivity properties of nanofluids can enhance the heat dissipation from MCHS in different microchannel structures. The effect of properties such as the surface tension and contact angle of nanofluids on MCHS cooling has been little investigated and this should be a key element of future research. It is necessary to explore in depth the coupling effect between each physical property of the nanofluid and the microchannel structure. The rapid miniaturization of electronic devices and the rapid development of molecular electronics are both placing higher demands on micro heat sinks; thus, improving the cooling performance of microchannels is urgent and needs to be looked at by a wide range of academics.

## Figures and Tables

**Table 1:** Thermal conductivity of common nanofluids.

Author	Nano Particles	Base Fluids	Mass Fraction (%)	Particle Size (nm)	Improvement of Thermal Conductivity
Aberoumand et al. [32]	Cu	Engine oil	0.2 0.5 1	50	27.0% 31.0% 49.0%
Kim [33,34]	Au	H_2_O	0.018	7–12	9.0%
Chen et al. [35]	Ag	H_2_O	0.1–0.3	10 80	18.0%
Arjmandfard et al. [36]	Fe	EG	0.20–0.55	10	13.0–18.0%
Li et al. [37]	CuO	H_2_O	0.025–0.1	45	3.0–13.5%
Hu et al. [38]	TiO_2_	H_2_O	3.85 7.41	10	2.12% 4.70%
Li et al. [39]	ZnO	75EG/25DW 85EG/15DW 95EG/5DW	5.25	30	8.00% 5.00% 3.00%
Wei et al. [40]	WO_3_	EG	0.005–5	<30	0.78–32.38%
Soltani et al. [41]	WO_3_/MWCNTs	Engine Oil	0.0–0.6	WO_3_: 23–65 MWCNT: 20–30	3.65–8.30%
Choi et al. [42]	Al_2_O_3_	EG/H_2_O	1.43	40–50	4.4%
Mei et al. [43]	Fe_3_O_4_	H_2_O	0.1 0.3 0.5	20	0.57% 1.75% 2.97%
Harandi et al. [44]	F-MWCNTs-Fe_3_O_4_	EG	0–2.3	20–30	30%
Pourrajab et al. [45]	MWCNTs–COOH-Ag	H_2_O	MWCNTs: 0.004–0.16 Ag: 0.04	MWCNTs: 20–30 Ag: 5–8	47.3%
Shahsavar et al. [46,47]	Fe_3_O_4_/CNT	H_2_O	Fe_3_O_4_: 0.5–0.9 CNT: 0.25–1.35	10	14.25– 58.74%
Tu et al. [48]	Fe_3_O_4_/AG	H_2_O	Fe_3_O_4_: 0.1–0.5 AG: 0.8	20	3.1–9.2%

**Table 2:** Optimization of cavity shape and structure.

Structure	Fluid	Conclusion
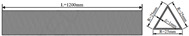 Details of the triangular tube [72]	TiO_2_-H_2_O	Qi et al. [72] found that the thermal properties of nanofluids in triangular tubes is superior to that of round tubes.
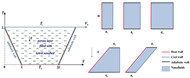 Cavity shape models [73,74]	Cu-Al_2_O_3_/H_2_O	Cimpean et al. [73,74] observed that trapezoidal and parallelogram cavities create dead zones in the flow of the nanofluids; therefore, rectangular chambers have a better heat-transfer capacity.
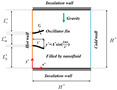 Cavity model with oscillating fins [75]	Nanofluids	Jamesahar et al. [75] discovered that the average *Nu* decreases with the increasing frequency of oscillation and increases with the increasing magnitude.
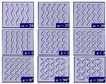 Waveform cavity model [76]	CuO-H_2_O	Tu et al. [76] concluded that for the same surface area, with increasing angular frequency and amplitude, the thermal performance is improved, the heat-transfer area rises, turbulence is enhanced and the thermal resistance of the boundary is reduced.

**Table 3:** Simulation of electric and magnetic fields and radiation on the flow properties of nanofluids.

Author	Conditions	Conclusion
Sardari et al. [78]	Electric field	At an electric field of 10 kV/m, the electrostatic force plays a dominant role, and at an electric field of 1 kV/m, the Brownian motion of the nanoparticles is more pronounced.
Sivaraj et al. [79] Fan et al. [80] Izadi et al. [81] Barnoon et al. [82]	Magnetic field	The greater the tilt angle of the magnetic field in the range 0–90°, the stronger the thermal properties of the nanofluids within the horizontal plate cavity. However, the effect of periodic magnetic fields on the thermal properties is not periodic.
Sheikholeslami et al. [83] Li et al. [84] Afrand et al. [85] Izadi et al. [86]	Thermal radiation and magnetic fields	The Nusselt number increases with increasing radiation parameters. The Nusselt number is greatest when the magnetic field is vertical. The nanofluids are subjected to both thermal radiation and magnetic fields, which both accelerates heat transfer and increases entropy production. The Lorentz force can affect the heat-transfer performance by changing the buoyancy force.

**Table 4:** Effect of ribs and cavities on the performance of MCHS.

Researchers	Rib/Cavity	Shape	Re	Comments
Kumar et al. [99]	Rib	Arc	200, 900, 2000	The MCHS with curved ribs produces a pseudo-secondary flow which enhances heat transfer. Structures with curved ribs on both the base plate and the side walls showed the greatest increase in *Nu*, by up to 119%, but at the expense of the Poiseuille number.
Derakhshanpour et al. [100]	Rib	Cylindrical	49–396	Reducing the rib spacing is more effective than increasing the radius of the cylindrical ribs in improving the performance of MCHS.
Li et al. [101]	Rib	Dual split-cylinder	50–300	The bottom dual-split-cylinder MCHS structure was proposed and optimized using an intelligent algorithm. Optimal dual-split-cylinder width decreases with increasing *Re*. For *Re* = 250, the best thermal performance of the optimized MCHS can be improved by 63.41%
Lori et al. [96]	Rib	Rectangle; Ellipse; Triangle	100–800	The best heat-transfer performance was achieved with triangular ribs at small heights and elliptical and rectangular ribs at large heights.
Wang et al. [97]	Rib	Truncated rectangle	100–1000	The *Nu* and surface friction coefficient increase as the rib width increases. Truncated ribs reduce the Δ*P*, which decreases with increasing truncated clearance, improving the comprehensive thermal performance.
Derakhshanpour et al. [102]	Rib	Semi-circular; Semi-elliptical	66–396	The curvature of the rib angles affects the heat-transfer performance of MCHS; the best performance is achieved with semi-circular ribs and filleted corner, with an increase in *Nu* of 18–21% and an increase in performance of 19–22%.
Qi et al. [103]	Cavity	Travelling wave	200–1000	Fractal MCHS with travelling wave cavities were found to improve the heat-transfer performance. The *Nu* increases and then decreases with increasing eccentricity, reaching a maximum at an eccentricity of 0.1.
Zhu et al. [104]	Cavity	Rectangle; Triangle; Trapezoid; Teardrop; Semicircle	194–610	The triangular-cavity MCHS shows the greatest improvement in thermal performance, but with a significant increase in Δ*P*, and the teardrop cavity MCHS has the best overall heat-transfer efficiency.
Huang et al. [105]	Rib or Cavity	Arc	200–1000	The ribbed MCHS has superior thermal performance, but also the a high Δ*P*. Combining thermal and hydraulic performance considerations, the cavity MCHS has the best performance.
Yao et al. [98]	Rib and Cavity	Triangular	0–600	Surface temperature is lowest at 0.0572 mm cavity height and 0.0224 mm rib height, which can be reduced by 17–26 K. Convective heat transfer dominates and flow friction increases with disturbance between the ribs.

**Table 5:** The effect of pin fins on the thermal and hydrological performance of MCHS.

Researchers	Structure	Shape	Re	Comments
Polat et al. [108]	Fin	Round, square, diamond	100–350	Diamond fins enhance thermal performance at the expense of hydraulic performance. Circular fins have the best performance; the best performance occurs when the porosity is large.
Vasilev et al. [109]	Fin	Circular	100–1000	The thermal and hydraulic performance of the PFMCHS increases with the height of the fin, with the optimum diameter and pitch being influenced by the height.
Ali et al. [110]	Fin	Rectangular, serrated	100–350	The serrated PFMCHS provides better performance, with a 6.44 K reduction in the average temperature of the heated surface, a 15% reduction in Δ*P* and a 60% increase in *Nu*.
Jia et al. [107]	Fin	Cone	147–637	When the pin fins are evenly distributed throughout the channel, the overall evaluation coefficient *η* is the largest.
Bhandari et al. [106]	Fin	Square	100–800	The 1.5 mm high pin fins open PFMCHS is the most efficient in terms of heat dissipation, when the convective surface area and open space are more conducive to enhancing the thermal performance.
Zeng et al. [111]	Fin	Open-ring	160–694	Open-ring pin fins cause fluid separation and convergence, and the flow boundary layer is interrupted periodically. The staggered open-ring PFMCHS offers the best overall performance.
Feng et al. [112]	Fin and cavity	[F]: Circular [C]: Rectangular	133–530	Two circular pin fins in the micro-cavity can effectively reduce the wall temperature and improve the temperature uniformity. The spacing of the fins affects the overall performance of the MCHS.
Rajalingam et al. [113]	Fin and blind hole	[F]: Circular, elliptical, aerofoil [B]: Circular	500–1000	MCHS with elliptical pin fins and circular blind holes has the best overall performance.
Bahiraei et al. [114]	Fin and rib	[F]: Trapezoidal, [R]: Rectangular	100–500	Changing the channel walls to trapezoidal fins that induce secondary flow reduces the increase in Δ*P* due to ribs. The *h* can be increased by 17% and the heat-transfer surface temperature can be reduced by up to 13.88 K.

**Table 6:** The influence of microchannel distribution on the thermal-hydraulic performance of the MCHS.

Structure	Working Fluids	Comments
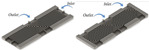 Heat sink A and heat sink B [120]	Graphene-silver nanofluid	Bahiraei et al. [120] found that as the nanofluids concentration and inlet velocity increase, the temperature and thermal resistance of the heating surface decreases and the temperature is more evenly distributed. Heat sink B has more intense fluid mixing and better heat transfer.
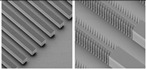 Rectangle and PW MCHS [121]	Pure acetone liquid	Jia et al. [121] revealed that the porous walls (PW) formed by the micro pin fin arrays on the side walls of the rectangular microchannels accelerate the liquid film disturbance, enhance evaporation heat transfer from the liquid film and improve the local heat coefficient.
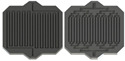 Serpentine and distributor MCHS [122]	GNP–silver nanofluid	Bahiraei et al. [122] discovered that the serpentine MCHS thermal performance is best when *Re* is constant, but the pumping power is high and the distributor-liquid-block thermal performance is best when the pumping power is constant.
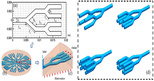 MCHS model for 3D fractals [123]	Deionised water	Tang et al. [123] proposed a three-dimensional fractal based on the disk fractal structure, and as the number of fractals increases, the surface temperature decreases by 2–4 K and the pressure drop increases by 5.96–8.29%. B-type topology has the best heat-transfer performance with a combined performance index of up to 1.29.
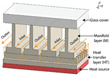 Model of MMCHS [124]	Water	Gilmore et al. [124] proposed a new open manifold microchannel heat sink (MMCHS) that can reduce its thermal resistance by optimizing the manifold shape. This MMCHS is derived to have a lower pressure drop of 25% and better overall performance.

**Table 7:** Flow and heat-transfer performance of different MCHS structures using nanofluids.

Author	Working Fluids	Structure	Comments
Sajid et al. [94]	TiO_2_-H_2_O	Wavy MCHS	The thermal performance of MCHS of TiO_2_-H_2_O nanofluid is better than that of distilled water, and its heat-dissipation performance decreases with increasing heating power.
Wu et al. [129]	Al_2_O_3_-H_2_O	RMCHS	The average temperature of the RMCHS heat-exchange surface decreases with increasing volume concentrations of nanoparticles, but there is a large increase in pumping power.
He et al. [130]	Al_2_O_3_-Cu-H_2_O	DL-MCHS	Average *Nu* of sinusoidal DL-MCHS increases with the increasing volume fraction of nanoparticles.
Duangthongsuk et al. [131]	SiO_2_-H_2_O	FMCHS CCZ-HS	The surface temperature of the circular fin MCHS (FMCHS) and the zigzag runner heat sink with a single cross-cut structure (CCZ-HS) decreases with increasing particle concentration.
Shamsuddin et al. [132]	BNNTs	RMCHS	At 50 °C, the pressure drop of MCHS is reduced by 3.2% and the heat-transfer performance is improved when the fluid is a mixture of surfactant TX-100 and 0.001wt% BNNTs compared to 0.001wt% BNNTs.
Ray et al. [133]	Al_2_O_3_/CuO/SiO_2_-EG/H_2_O (6:4)	RMCHS	CuO-EG/H_2_O (6:4) offers the most significant performance improvement, with a 21% increase in convective heat-transfer coefficient and 22% reduction in pumping power.
Noh et al. [134]	CuO/Diamond/Al_2_O_3_/SiO_2_-H_2_O	RMCHS	The use of Diamond-H_2_O nanofluids as a coolant results in a 0.3% increase in heat-dissipation efficiency.
Shahsavar et al. [135]	Fe_3_O_4_-CNT-H_2_O	Double-pipe MCHS	The thermal characteristics of the non-Newtonian Fe_3_O_4_-CNT-H_2_O nanofluids are higher than those of Newtonian Fe_3_O_4_-CNT-H_2_O nanofluids, while the flow characteristics such as pressure drop and pump power are the opposite.
Muallim et al. [136]	Al_2_O_3_/CuO-H_2_O/EG/PAO	RMCHS with longitudinal vortex generations	The CuO-PAO (Polyalphaolefin) nanofluid has the best performance, with a *Nu* of 15.88, and this fluid has the best performance in RMCHS with longitudinal vortex generation.
Wang et al. [137]	Al_2_O_3_-H_2_O DI-H_2_O	FMCHS with vortex generators	The best heat-transfer performance of the FMCHS is achieved at a particle size of 20 nm and a volume fraction of 4% for Al_2_O_3_ nanoparticles.

## Data Availability

Not applicable.
